# Individual diseases or clustering of health conditions? Association between multiple chronic diseases and health-related quality of life in adults

**DOI:** 10.1186/s12955-017-0806-6

**Published:** 2017-12-21

**Authors:** David Alejandro González-Chica, Catherine L. Hill, Tiffany K. Gill, Phillipa Hay, Dandara Haag, Nigel Stocks

**Affiliations:** 1 Discipline of General Practice, Adelaide Medical School, NHMRC Centre of Research Excellence to Reduce Inequality in Heart Disease, The University of Adelaide, Hughes Building, Level 8, Room 813, Adelaide, SA 5005 Australia; 2 Department of Rheumatology, The Queen Elizabeth Hospital, Adelaide, SA Australia; 3 Discipline of Medicine, Adelaide Medical School, The University of Adelaide, Adelaide, SA Australia; 4 Centre for Health Research, School of Medicine, Western Sydney University, Sydney, NSW Australia; 5 Australian Research Centre for Population Oral Health, School of Dentistry, The University of Adelaide, Adelaide, SA Australia

**Keywords:** Quality of life, Multimorbidity, Multiple chronic conditions, Chronic disease, Epidemiologic methods

## Abstract

**Background:**

Chronic diseases are highly prevalent and cluster in individuals (multimorbidity). This study investigated the association between multimorbidity and Health-Related Quality of Life (HRQoL), assessing the combination of chronic diseases highly correlated with this outcome.

**Methods:**

We conducted a household survey in 2015 in a random sample of 2912 South Australian adults (48.9 ± 18.1 years; 50.9% females), obtaining information on sociodemographics, lifestyle, and 17 chronic conditions clustered in four different groups (metabolic, cardiovascular, gastrointestinal, and musculoskeletal). Information on physical (PCS) and mental components scores (MCS) of HRQoL were assessed using the SF-12 questionnaire. Multivariable linear regression models considering individual diseases (mutually adjusted) and clusters within- and between-groups were used to test the associations.

**Results:**

Only 41% of the sample was negative for all the investigated diseases. The most prevalent conditions were osteoarthritis, obesity and hypertension, which affected one in every four individuals. PCS was markedly lower among those reporting stroke, heart failure, and osteoarthritis, but they were not associated with MCS. Direct-trend relationships were observed between the number of chronic conditions (clusters within- and between-groups) and PCS, but not with MCS. The strongest association with PCS was for musculoskeletal conditions (difference between those affected by 2+ conditions and those free of these conditions −6.7 95%CI -8.5;-5.4), and lower PCS were observed in any combination of clusters between-group including musculoskeletal diseases.

**Conclusion:**

In the context of multimorbidity, musculoskeletal diseases are a key determinant group of PCS, amplifying the association of other chronic conditions on physical but not on mental health.

## Background

Non-communicable chronic diseases (NCDs) are the leading cause of mortality, being responsible for 68% of all deaths (38 million) worldwide in 2012. The proportional mortality related to NCDs is greater in high-income countries, where they account for 87% of all deaths, compared to 57% and 37% in middle- and low-income countries, respectively [1]. However, there is a considerable variability in the proportion of deaths attributable to NCDs among high-income countries, ranging from less than 80% in Asiatic countries to more than 90% in some European countries (Finland, Switzerland, Germany, Italy) and Australia [2]. Moreover, NCDs are also related to a substantial burden for individuals and society, due to their long-lasting effect on health status, frequent health care utilisation, loss of productivity, and reduced health-related quality of life (HRQoL) [3–5].

HRQoL is a patient-centered outcome that assesses the impact of health conditions on daily living, based on the self-perception of the individuals, and considers their social and cultural context [4, 6]. Several studies performed in clinical settings have reported different NCDs negatively affect HRQoL [4]. However, few population-based studies have examined whether some combinations of NCDs are more correlated with this outcome [7, 8]. To investigate the relationship between multimorbidity (clusters of different NCDs affecting the same individual) [5] and the different domains of HRQoL is particularly relevant for public health purposes, considering this outcome is closely related to the adherence to health management, hospitalisations, and mortality [6, 9]. Furthermore, in the last decades, there has been a progressive rise in the prevalence of NCDs and multimorbidity [2, 3]. Although the increase in life expectancy is one of the reasons for this change, other aspects seem to contribute to multimorbidity. Firstly, various NCDs share similar risk factors, such as smoking, inadequate diets, excessive alcohol intake, and stress [5], and some of them have also increased in the last few years [2, 3]. Secondly, unhealthy habits are more likely to occur together, increasing the vulnerability to multiple health conditions [10]. Finally, NCDs tend to generate an overall state of immune suppression and/or inflammation, increasing the risk of developing other chronic diseases [5].

Understanding the relationship between clusters of NCDs and HRQoL could assist health policy makers to identify chronic disease combinations highly correlated with this outcome, as they might be useful for developing preventive strategies and improving clinical guidelines [6, 9, 11]. This is particularly relevant considering that clinical guidelines are usually created for the management of specific diseases and rarely account for multimorbidity [12]. The aim of this study was to investigate the association between chronic health conditions (individual diseases and clusters within- and between-groups) and HRQoL (physical and mental domains) among adults living in South Australia.

## Methods

This is a cross-sectional study using face-to-face interviews, including a representative sample of adults living in South Australia (SA) in 2015. SA has approximately 1.7 million inhabitants (73% living in the capital), a life expectancy of 83.0 years, and a very high human development index of 0.907 (composite index of life expectancy, education, and per capita income indicators), equivalent to that of Sweden and the UK [13].

Details of the methodology have been published elsewhere [14, 15]. In summary, a multistage sampling process was used to select individuals. Firstly, cities/localities were stratified according to their location (metropolitan or rural). Secondly, 530/3939 Statistical Areas Level 1 (SA1 i.e. the smallest division unit used in the Australian census in 2011) [13] were systematically selected (selection probability proportional to their size). Thirdly, clusters of 10 residences were systematically selected in each SA1. Finally, one dweller aged 15+ years was randomly chosen (last person to have a birthday) in each of the selected households. Individuals were excluded if terminally ill/mental incapacitated (*n* = 104) or unable to speak English (*n* = 87). Of the 4226 eligible participants, 1221 refused to answer the survey, providing a final sample of 3005 individuals (71.1%). For the purposes of this paper, only individuals aged 20+ years were included in the analyses (*N* = 2912).

### Patient-centered outcome

The patient-centered tool used in this study was the Medical Outcomes Study Short Form 12 (SF-12v1). The 12 questions in this instrument evaluate HRQoL in the past four weeks, generating two different 0–100 component scores (physical (PCS) and mental (MCS)), with higher values indicating a better HRQoL [16–18]. The SF-12v1 is an instrument with good psychometric properties, including test-retest reliability coefficients of 0.9 for PCS and 0.8 for MCS, and appropriate construct validity (relative validity of 0.93 for PCS and 1.1 for MCS in the discrimination of groups of patients who differ in physical and mental health according to proven clinical measures) [18].

### Multimorbidity and clusters of chronic conditions

The diagnosis of clinical conditions was based on the medical diagnosis (“*have you ever been told by a doctor that you have…*”) and/or treatment (“*are you on medication for*…”) for 17 chronic conditions, classified as 1) metabolic (obesity, diabetes, dyslipidaemia); 2) cardiovascular (hypertension, myocardial infarction, angina, heart failure, atrial fibrillation/arrhythmia, stroke); 3) gastrointestinal (gastroesophageal reflux, irritable bowel syndrome, Crohn’s disease, ulcerative colitis, and/or coeliac disease), and; 4) musculoskeletal (osteoarthritis, gout, osteoporosis). Obesity was defined as a body mass index ≥30 kg/m^2^ [19] and investigated based on self-reported weight and height.

Clustering within-group was assessed by counting the number of health conditions in each group (metabolic, cardiovascular, gastrointestinal, and musculoskeletal). For the clusters between-group, the counts of chronic conditions within each group were transformed into binary variables (none or 1+ condition in the correspondent group). The four binary variables were then combined to create 16 possible combinations: negatives for all conditions, positives for just one group (4 possibilities), two-group clusters (6 possible combinations), three-group clusters (4 possible combinations), and positives for all the groups.

### Confounding variables

Sociodemographic, lifestyle, and mental health status variables were included as possible confounders of the tested associations, as they are associated with the occurrence of chronic conditions and correlated with HRQoL [20–23]. These variables included gender (male or female), age (in years, including a quadratic term for non-linear associations), marital status (married/living with a partner - yes or no), residence area (urban or rural), attained educational level (bachelor or higher; trade qualification; certificate/diploma; secondary; less than secondary), working status (employed full-time; employed part-time; unemployed; retired), and macro-level socioeconomic position. Macro-level socioeconomic position was investigated using the 2011 Australian Socio-Economic Indexes for Areas Index of Relative Socio-economic Advantage and Disadvantage (SEIFA-IRSAD). This index is based on a range of census variables and is an indicator of relative economic and social advantage/disadvantage of people and households within an area [24], with high scores indicating the respondent residing in a more advantaged area.

Lifestyle variables included daily fruit/vegetable consumption (0–2, 3–4, or 5+ portions/day), weekly practice of 30+ minutes of moderate/vigorous physical activity (0–1, 2–4, or 5+ days/week), daily alcohol consumption (0–2, 3–4, or 5+ standard drinks/day), and smoking status (never, former, or current smoker). Individuals were considered positives for a mental health condition when they referred receiving treatment for anxiety, depression, or any other mental health problem. Mental health status (*“currently receiving treatment for anxiety, depression, or any other health problem”*) was also included as a possible confounder, considering the associations could be overestimated among individuals affected by these conditions [20, 25].

### Data analysis

Categorical variables were described considering absolute and relative frequencies (%), while mean and standard deviation or median with interquartile range (p25-p75) were used for numerical variables, depending on their symmetry.

Linear regression models were used to evaluate the association between chronic conditions (individual, cluster within-groups, and clusters between-groups) and HRQoL. The results were fully adjusted for sociodemographic variables, lifestyle, and mental health status, independently of their *p*-value in the association with the outcomes (Model 1). Additionally, individual chronic conditions and clusters within-groups were mutually adjusted (Model 2), to take into account the coexistence of the other conditions in the same individual (multimorbidity). Regression coefficients (β) or marginal adjusted means of HRQoL with their respective confidence intervals of 95% (95%CI) were estimated. An alpha of 5% was defined as indicative of statistical significance. Determination coefficients (R^2^) were used to evaluate the overall model fit, while the variance inflation factor (VIF) was investigated as an indicator of possible collinearity between the explanatory variables. [26]

All the analyses were performed in STATA 14.0 (StataCorp, Texas, USA), weighted to the inverse of the individual’s probability of selection within the household, re-weighted to the estimated resident population in SA in 2014 (according to age and sex), and analyzed considering the sampling design (clusters of SA1) [13, 14]. For the analysis of clusters between-group, an additional weight was considered, to account for the number of chronic conditions in each group (probability between-group multiplied by the probability of each condition within the correspondent group).

This study was approved by the University of Adelaide Human Research Ethics Committee (project H-097-2010) and participants provided their consent to participate in the survey.

## Results

The mean age of the 2912 individuals included in the analyses was 48.9 ± 18.1 years (50.9% females). Participants’ sociodemographic characteristics and lifestyle variables are described in Table 1. The mean PCS (48.4 ± 10.4 points) was lower than MCS (52.4 ± 8.8 points).

**Table 1:** Sample distribution according to sociodemographic variables among individuals ≥20 years in South Australia, 2015 (unweighted *N* = 2912)

Variables	Number of participants (%)
Gender: Females	1482 (50.9)
Age group	
20–34 years	789 (27.1)
35–49 years	719 (24.7)
50–64 years	757 (26.0)
65–79 years	494 (17.0)
≥ 80 years	153 (5.3)
Marital status: Married/living with a partner	1942 (66.7)
Residence area: Urban	2169 (74.5)
Educational level	
Bachelor or higher	743 (25.5)
Trade qualification	384 (13.2)
Certificate/diploma	821 (28.2)
Secondary	705 (24.2)
Less than secondary	259 (8.9)
Working status	
Employed full time	1083 (37.2)
Employed part time	594 (20.4)
Not working	577 (19.8)
Retired	658 (22.6)
Socioeconomic position (quartiles)^a^	
High	818 (28.1)
Middle-high	646 (22.2)
Middle-low	725 (24.9)
Low	722 (24.8)
Fruit/vegetable consumption (portions/day)	
0–2	842 (28.9)
3–4	1141 (39.2)
5+	929 (31.9)
Physical activity (days/week)	
0–1	864 (29.7)
2–3	1050 (36.1)
5+	998 (34.3)
Alcohol intake (doses/day)	
0–2	1902 (65.3)
3–4	597 (20.5)
5+	413 (14.2)
Smoking status	
Never	1226 (42.1)
Former	1229 (42.2)
Current	457 (15.7)

^a^Based on the Socio-Economic Indexes for Areas Index of Relative Socio-economic Advantage and Disadvantaged (SEIFA-IRSAD)

The weighting of data can lead to rounding effects and totals not adding to 100%

Table 2 presents the prevalence of the 17 independent chronic conditions (divided into four groups) and their association with HRQoL. The most prevalent conditions were osteoarthritis, obesity and hypertension, which affected one in every four individuals. In crude analysis, all the conditions were related to a lower PCS, although the results for ulcerative colitis, Crohn’s disease, and coeliac disease were non-significant. After adjustment for sociodemographic, lifestyle and mental health status variables (Model 1), there was a considerable reduction in the magnitude of the associations (at least 50% reduction for most of the associations), but the statistical significance remained stable. A further reduction of the differences between categories was observed when the chronic conditions were mutually adjusted (Model 2), and only nine conditions remained associated with a lower PCS: obesity, diabetes, atrial fibrillation, stroke, heart failure, gastroesophageal reflux, irritable bowel syndrome, osteoarthritis, and osteoporosis. PCS was markedly lower among those reporting stroke, heart failure, and osteoarthritis. Regarding MCS, only six conditions were associated with a lower score: obesity, hypertension, gastroesophageal reflux, irritable bowel syndrome, and ulcerative colitis. After adjustment for covariates and mutual adjustment for other health conditions (Model 2), only ulcerative colitis, irritable bowel syndrome, and osteoporosis remained related to a lower MCS, with ulcerative colitis showing the strongest association.

**Table 2:** Association between metabolic, cardiovascular, gastrointestinal, and musculoskeletal conditions with the health related quality of life among individuals ≥20 years in South Australia, 2015 (N = 2912)

	%	Physical component score			Mental component score		
		Crude	Adjusted		Crude	Adjusted	
			Model 1	Model 2		Model 1	Model 2
		Mean difference (SE)	Mean difference (SE)	Mean difference (SE)	Mean difference (SE)	Mean difference (SE)	Mean difference (SE)
Metabolic conditions							
Obesity*	25.3	-5.2 (0.5)^a^	−3.6 (0.5)^a^	−2.3 (0.4)^a^	−0.9 (0.4)^c^	−0.3 (0.4)	0.0 (0.4)
Dyslipidaemia	16.9	−7.3 (0.6)^a^	−2.5 (0.6)^a^	−0.3 (0.6)	0.3 (0.5)	0.0 (0.4)	0.0 (0.5)
Diabetes	9.3	−7.8 (0.7)^a^	−3.7 (0.6)^a^	−2.2 (0.6)^b^	−0.6 (0.6)	−0.1 (0.5)	−0.2 (0.5)
Cardiovascular conditions							
Hypertension	22.4	−7.6 (0.6)^a^	−2.8 (0.7)^a^	−1.1 (0.6)	1.3 (0.4)^b^	0.8 (0.4)	1.0 (0.5)
Heart attack	5.2	−9.4 (1.0)^a^	−3.7 (1.0)^a^	−1.7 (1.2)	0.4 (0.7)	−0.8 (0.7)	−0.9 (0.8)
Atrial fibrillation	3.2	−8.4 (1.2)^a^	−4.0 (1.1)^a^	−2.7 (1.0)^b^	0.9 (0.9)	−0.1 (0.9)	0.1 (0.9)
Angina	2.3	−12.1 (1.5)^a^	−5.2 (1.3)^a^	−1.7 (1.2)	−1.1 (1.3)	−0.4 (1.1)	0.2 (1.2)
Stroke	1.7	−13.0 (1.3)^a^	−6.5 (1.6)^a^	−4.2 (1.7)^c^	−1.7 (1.5)	−1.3 (1.5)	−1.3 (1.5)
Heart failure	1.0	−14.9 (1.6)^a^	−9.1 (1.6)^a^	−6.1 (1.5)^a^	1.0 (1.4)	−0.2 (1.3)	0.2 (1.4)
Gastrointestinal conditions							
Gastroesophageal reflux	8.4	−6.1 (0.9)^a^	−3.3 (0.7)^a^	−1.7 (0.7)^c^	−2.3 (0.8)^b^	−1.1 (0.7)	−0.8 (0.7)
Irritable bowel syndrome	7.9	−4.7 (0.9)^a^	−2.9 (0.7)^a^	−1.7 (0.7)^c^	−3.3 (0.7)^a^	−1.9 (0.6)^b^	−1.7 (0.6)^b^
Ulcerative colitis	0.9	−5.0 (2.6)	−2.2 (2.2)	−0.9 (1.8)	−6.0 (1.7)^b^	−5.0 (1.7)^b^	−4.5 (1.7)^b^
Crohn’s disease	0.8	−4.7 (3.1)	−3.7 (2.7)	−3.0 (2.5)	−2.4 (2.4)	−1.2 (1.8)	−0.1 (1.6)
Coeliac disease	0.7	−1.7 (2.1)	−1.9 (1.6)	−1.7 (1.9)	−0.6 (0.6)	−0.1 (1.1)	0.4 (1.1)
Musculoskeletal conditions							
Osteoarthritis	25.6	−10.4 (0.5)^a^	−7.1 (0.5)^a^	−6.1 (0.5)^a^	−0.6 (0.5)	0.0 (0.5)	0.2 (0.5)
Gout	6.1	−5.3 (0.9)^a^	−1.9 (0.9)^c^	−0.1 (0.9)	0.3 (0.7)	−0.9 (0.6)	−0.8 (0.6)
Osteoporosis	5.2	−8.7 (0.9)^a^	−3.1 (0.8)^a^	−2.1 (0.7)^b^	−1.2 (0.8)	−1.4 (0.7)	−1.5 (0.7)^c^

*Obesity was defined as having a BMI ≥ 30, based on self-reported data for weight and height

Model 1: predicted means adjusted for sex, age, marital status, area of residence, educational level, working status, socioeconomic position (Socio-Economic Indexes for Areas Index of Relative Socio-economic Advantage and Disadvantaged), fruit/vegetable consumption, physical activity, smoking status, alcohol intake, and mental health status

Model 2: predicted means adjusted for variables in model 1 + mutual adjustment between chronic conditions

Associations with a p-value a < 0.001; b < 0.01; c < 0.05

Crude R^2^ for PCS and MCS were: Obesity 4.8% and 0.2%; Dyslipidaemia 7.0% and 0.02%; Diabetes 4.7% and 0.04%; Hypertension 9.2% and 0.4%; Heart attack 4.0% and 0.01%; Atrial fibrillation 2.0% and 0.03%; Angina 3.1% and 0.03%; Stroke 2.6% and 0.1%; Heart failure 2.0% and 0.01%; Gastroesophageal reflux 2.7% and 0.5%; Irritable bowel syndrome 1.5% and 1.1%; Ulcerative colitis 0.2% and 0.4%; Chron’s disease 0.2% and 0.1%; Coeliac disease 0.02% and 0.01%; Osteoarthritis 19.2% and 0.1%; Gout 1.5% and 0.01%; Osteoporosis 3.5% and 0.1%. Adjusted R^2^ for models including just sociodemographic, lifestyle, and mental health status variables were 27.2% for PCS and 23.4% for MCS, while R^2^ for full models with mutual adjustment of all conditions (Model 2) were 38.1% and 24.4%, respectively

Table 3 displays the frequency of clustering within-groups and their association with HRQoL. Clusters within-group (positives for 2+ conditions of the same group) were more frequent for metabolic conditions (10.7%), followed by cardiovascular (6.9%), musculoskeletal (6.1%), and gastrointestinal conditions (2.1%). An inverse trend association was observed between the number of chronic conditions in each group and PCS in crude and adjusted analysis. In the fully adjusted model (Model 2), the largest difference was for musculoskeletal conditions (difference between those affected by 2+ conditions and those free of these conditions −6.7 95%CI -8.5;-5.4), followed by cardiovascular (−4.6 95%CI -6.5;-2.7), gastrointestinal (−3.7 95%CI -6.3;-1.1), and metabolic conditions (−3.1 95%CI -4.5;-1.8). For MCS, in Model 2 only gastrointestinal conditions remained related to a lower mental score (−3.4 95%CI -5.6;-1.2).

**Table 3:** Association between clusters intra-groups of metabolic, cardiovascular, cardiovascular, and musculoskeletal conditions with the health related quality of life among individuals ≥20 years in South Australia, 2015 (*N* = 2912)

		Physical component score				Mental component score	
	(%)	Crude	Adjusted		Crude	Adjusted	
			Model 1	Model 2		Model 1	Model 2
		Mean (SE)	Mean (SE)	Mean (SE)	Mean (SE)	Mean (SE)	Mean (SE)
Metabolic conditions		p < 0.001	p < 0.001	p < 0.001	*p* = 0.21	*p* = 0.99	*p* = 0.90
None	61.4	51.1 (0.3)	49.9 (0.2)	49.4 (0.2)	52.5 (0.2)	52.4 (0.2)	52.4 (0.2)
1	27.9	45.3 (0.4)	46.5 (0.4)	47.1 (0.3)	52.4 (0.4)	52.5 (0.3)	52.4 (0.3)
2+	10.7	40.8 (0.7)	44.7 (0.6)	46.3 (0.6)	51.7 (0.5)	52.4 (0.4)	52.3 (0.4)
*R* ^*2*^		*12.4%*	*30.2%*	*36.9%*	*0.1%*	*23.4%*	*24.1%*
Cardiovascular conditions		p < 0.001	p < 0.001	p < 0.001	*p* = 0.02	*p* = 0.51	*p* = 0.39
None	74.0	50.5 (0.2)	49.4 (0.2)	49.0 (0.2)	52.1 (0.2)	52.3 (0.2)	52.3 (0.2)
1	19.1	44.1 (0.6)	46.8 (0.6)	47.8 (0.5)	53.3 (0.7)	53.1 (0.3)	53.1 (0.4)
2+	6.9	37.9 (0.8)	42.4 (0.9)	44.3 (0.9)	52.8 (1.1)	52.1 (0.6)	52.3 (0.6)
*R* ^*2*^		*13.2%*	*29.5%*	*36.9%*	*0.3%*	*23.5%*	*24.1%*
Gastrointestinal conditions		p < 0.001	p < 0.001	p < 0.001	p < 0.001	p < 0.001	*p* = 0.001
None	83.6	49.2 (0.3)	48.9 (0.2)	48.7 (0.2)	52.8 (0.2)	52.7 (0.2)	52.7 (0.2)
1	14.3	44.7 (0.6)	46.2 (0.5)	46.7 (0.5)	50.7 (0.5)	51.6 (0.5)	51.6 (0.5)
2+	2.1	44.7 (1.6)	43.2 (1.3)	45.0 (1.3)	47.1 (1.5)	48.7 (1.1)	49.2 (1.1)
*R* ^*2*^		*3.8%*	*28.4%*	*36.9%*	*1.5%*	*23.9%*	*24.1%*
Musculoskeletal conditions		p < 0.001	p < 0.001	p < 0.001	*p* = 0.18	*p* = 0.16	*p* = 0.34
None	69.5	51.4 (0.2)	50.5 (0.2)	50.2 (0.2)	52.6 (0.2)	52.5 (0.2)	52.5 (0.2)
1	24.4	42.1 (0.4)	44.0 (0.4)	44.6 (0.4)	52.1 (0.5)	52.4 (0.4)	52.5 (0.4)
2+	6.1	38.9 (0.8)	42.1 (0.8)	43.2 (0.7)	51.7 (0.8)	51.2 (0.7)	51.4 (0.7)
*R* ^*2*^		*20.1%*	*33.6%*	*36.9%*	*0.1%*	*23.5%*	*24.1%*

Metabolic conditions: obesity, dyslipidaemia, and/or diabetes mellitus. Cardiovascular conditions: hypertension, myocardial infarction, angina, atrial fibrillation, heart failure, and/or stroke. Gastrointestinal conditions: irritable bowel syndrome, gastroesophageal reflux, coeliac disease, Chrohn’s disease and/or ulcerative colitis. Musculoskeletal conditions: osteoarthritis, gout and/or osteoporosis

Model 1: predicted means adjusted for sex, age, marital status, area of residence, educational level, working status, socioeconomic position (Socio-Economic Indexes for Areas Index of Relative Socio-economic Advantage and Disadvantaged), fruit/vegetable consumption, physical activity, smoking status, alcohol intake, and mental health status

Model 2: predicted means adjusted for variables in model 1 + mutual adjustment between the cluster intragroup of chronic conditions

R^2^ = Determination coefficient in %. Adjusted R^2^ for models including just sociodemographic, lifestyle, and mental health status variables were 27.2% for PCS and 23.4% for MCS. The R^2^ for Model 2 is the same for all clusters intragroup, as the regression models included a mutual adjustment between them

Table 4 shows the prevalence of clusters between-groups and their relationship with HRQoL. Only 41.0% of the participants were negative for all the investigated conditions, 25.2% were positive for only one group, and 3.1% were positive for the four groups. The most common two-group combinations were metabolic + cardiovascular (5.7%) and metabolic + musculoskeletal (5.5%), while for the three-group cluster the most frequent was metabolic + cardiovascular + musculoskeletal (8.3%). For PCS, in general, the higher the number of chronic conditions, the lower the score, both in crude and adjusted analysis (*p* < 0.001). Moreover, musculoskeletal conditions were a key group in these associations, as a lower PCS was observed in all clusters involving these diseases. PCS was 11.3 points lower (95%CI -14.0;-8.7) among those positives for all four groups of chronic conditions than among those negatives for all of them. On the other hand, no any specific pattern was observed for the association with MCS. The cluster gastrointestinal + musculoskeletal was the only combination associated with a lower score (−2.6 95%CI -5.2;0.0; *p* = 0.013) when compared to those negative for all the chronic conditions.

**Table 4:** Association between clusters between-groups of metabolic, cardiovascular, cardiovascular, and musculoskeletal conditions with the health related quality of life among individuals ≥20 years in South Australia, 2015

	%	Physical component score		Mental component score	
		Crude	Adjusted	Crude	Adjusted
		Mean (SE)	Mean (SE)	Mean (SE)	Mean (SE)
No disease	41.0	52.9 (0.3)	52.7 (0.3)	52.5 (0.3)	52.6 (0.2)
One group only					
Metabolic conditions	11.0	50.0 (0.5)	50.4 (0.5)	52.5 (0.6)	52.7 (0.5)
Cardiovascular conditions	3.3	50.3 (0.8)	51.5 (0.8)	53.8 (0.7)	52.5 (0.7)
Gastrointestinal conditions	4.6	50.5 (0.9)	50.1 (0.9)	50.6 (0.9)	51.2 (0.9)
Musculoskeletal conditions	6.3	46.0 (0.8)	47.0 (0.8)	52.4 (0.7)	52.5 (0.6)
Two-group clusters					
Metabolic + Cardiovascular	5.7	46.4 (0.9)	48.4 (0.9)	53.7 (0.7)	52.6 (0.7)
Metabolic + Gastrointestinal	1.9	47.6 (1.3)	48.7 (1.6)	49.2 (1.6)	51.3 (1.1)
Metabolic + Musculoskeletal	5.5	42.0 (1.0)	43.7 (0.9)	49.8 (0.9)	50.7 (0.7)
Cardiovascular + Gastrointestinal	1.0	48.9 (1.9)	50.0 (1.6)	52.3 (2.2)	52.1 (2.1)
Cardiovascular + Musculoskeletal	2.6	44.9 (1.2)	47.9 (1.1)	54.3 (0.7)	52.8 (0.7)
Gastrointestinal + Musculoskeletal	2.0	42.6 (1.5)	44.2 (1.4)	49.2 (1.4)	49.9 (1.3)
Three-group clusters					
Metabolic + Cardiovascular + Gastrointestinal	1.4	44.3 (1.4)	46.7 (1.3)	52.5 (1.3)	51.5 (1.2)
Metabolic + Cardiovascular + Musculoskeletal	8.3	39.0 (0.7)	41.8 (0.7)	54.8 (0.5)	53.5 (0.6)
Metabolic + Gastrointestinal + Musculoskeletal	1.7	39.7 (1.3)	41.8 (1.4)	48.5 (1.5)	51.2 (1.3)
Cardiovascular + Gastrointestinal + Musculoskeletal	1.0	39.1 (1.6)	42.6 (1.5)	50.4 (1.8)	50.7 (1.8)
Positives for the four groups	3.1	38.2 (1.3)	41.3 (1.3)	53.0 (1.1)	53.6 (1.1)
*R* ^*2*^		*27.1%*	*36.7%*	*2.5%*	*24.2%*

Metabolic conditions: obesity, dyslipidaemia, and/or diabetes mellitus. Cardiovascular conditions: hypertension, myocardial infarction, angina, atrial fibrillation, heart failure, and/or stroke. Gastrointestinal conditions: irritable bowel syndrome, gastroesophageal reflux, coeliac disease, Crohn’s disease and/or ulcerative colitis. Musculoskeletal conditions: osteoarthritis, gout and/or osteoporosis

R^2^ = Determination coefficient in %. Adjusted R^2^ for models including just sociodemographic, lifestyle, and mental health status variables were 27.2% for PCS and 23.4% for MCS

Model 1: predicted means adjusted for sex, age, marital status, area of residence, educational level, working status, socioeconomic position (Socio-Economic Indexes for Areas Index of Relative Socio-economic Advantage and Disadvantaged), fruit/vegetable consumption, physical activity, smoking status, alcohol intake, and mental health status

Model 2: predicted means adjusted for variables in model 1 + mutual adjustment between the cluster intragroup of chronic conditions

Compared to the model including just sociodemographic, lifestyle, and mental health status variables (adjusted R^2^ = 27.2%), there was an increment of 35–40% in the variability of PCS explained by the chronic conditions, with small differences between models including individual chronic conditions (adjusted R^2^ = 38.1%), clusters within-group (adjusted R^2^ = 36.9%), or clusters between-group (adjusted R^2^ = 36.7%). For the mental HRQoL, the adjusted R^2^ for the regression model including just the covariates (23.4%) remained relatively stable after including the chronic conditions (24.4%, 24.1%, and 24.2%, respectively). No evidence of multicollinearity between the explanatory variables was identified, as the mean VIF did not exceed 1.62 in any model.

## Discussion

To our knowledge, this is the first study investigating multimorbidity using clinically relevant clusters of chronic conditions. Four main findings can be highlighted based on our results. Firstly, chronic conditions, either individually or in clusters (within- or between-group), were consistently associated with a lower PCS, but the relationship with MCS was subtle. Secondly, regarding individual diseases, the lowest PCS was observed among those with stroke, heart failure and osteoarthritis; better management of osteoarthritis could therefore have a positive public health impact, considering its reported high prevalence. Thirdly, direct-trend relationships were observed between the number of chronic conditions (clusters within- and between-groups) and PCS. Finally, musculoskeletal conditions are particularly relevant in these associations, not only because they showed the lowest PCS among the clusters within-group, but because lower scores were also observed in any combination of clusters between-group including musculoskeletal diseases.

The negative correlation between multimorbidity and PCS but not with MCS observed in our study is consistent with the findings of a systematic review published in 2004 [4]. All of the 27 studies included in that review found an inverse trend relationship between the number of chronic conditions and PCS, but the association with MCS was inconclusive. Additionally, a study involving representative samples from eight high-income countries (Denmark, France, Germany, Italy, Japan, The Netherlands, Norway and the United States) investigated from 1990 to1996, reported that chronic diseases had a stronger association with PCS than with MCS [27]. In agreement with our results, arthritis and heart failure were two of the conditions highly correlated with PCS (−4.5 and −4.4, respectively). More recent population-based studies conducted in different income settings and aiming to investigate the relationship between multimorbidity and HRQoL identified similar findings [9, 28]. Therefore, the increasing prevalence of chronic diseases [1, 29] in SA during the last two decades could explain the progressive reduction in PCS [from 49.8 (95%CI 49.4–50.2) in 1997 to 48.6 (95%CI 48.2–49.0) in 2015], while MCS has remained constant [30]. This hypothesis should be explored in future longitudinal studies.

These results suggest new challenges for the Australian health care system, considering HRQoL is closely related to the adherence to health management, hospitalisations, and mortality among individuals affected by chronic conditions [6, 9]. Health indicators for Australia are strong and the country has one of the longest life expectancies among high-income economies, largely due the universal access to a comprehensive range of services (i.e. general practice, community and emergency health services, hospital care, rehabilitation and palliative care, prescriptions) provided by Medicare and the national health care system [13, 31]. Nevertheless, similar to other developed countries, there are concerns about the future of this publicly funded health service, considering the ageing of the population, rising levels of obesity, and the increasing burden of chronic conditions [1, 29, 31].

In this sense, among the investigated chronic diseases, musculoskeletal conditions seem to be a key group that determines PCS. When investigating clusters within-group, the regression coefficients for the association between these conditions and PCS were 46% higher than for cardiovascular diseases, and twice the value of gastrointestinal and metabolic conditions. For the clusters between-group, PCS was at least four points lower in all combinations including musculoskeletal conditions, compared to those combinations without them. These results are consistent with the findings of two surveys conducted in Spain (1999–2000) [32] and the Netherlands (2010) [33]. According to these studies, musculoskeletal conditions amplified the negative association between multimorbidity and PCS but not with MCS, irrespective of the number of other chronic conditions. These findings are particularly relevant in terms of public health, considering musculoskeletal diseases affect between 10%–35% of the population over the age of 35 years, and have become a leading cause for years lived with disability (YLDs) and Disability-adjusted life years (DALYs) in the last 30 years [34, 35]. Additionally, musculoskeletal conditions are responsible for a two-fold increase in healthcare costs, which is the second highest increase after cancer [33].

Finally, although the assessment of the associations between multimorbidity and HRQoL (variation in the scores based on the R^2^) was better explained in the model including the individual conditions than the clusters, within- or between-group, the differences in R^2^ were small. Furthermore, the latter approach considering all the combinations of clusters between-group seems more relevant for clinical practice, as it allows identification of the combinations most strongly associated with a lower HRQoL.

Some limitations should be discussed. Firstly, the diagnosis of chronic health conditions and behavioural risk factors was by self-report. Although reliable and highly specific [36, 37], the validity of this information is reduced by intermediate levels of sensitivity (ranging from 33 to 85%) [37]. However, this possible source of bias is less likely to explain our results, as it would have reduced the magnitude of the associations. Secondly, the assessment of HRQoL was probably compromised among individuals with mental health conditions, as their judgment regarding subjective outcomes, such as well-being and/or satisfaction with their life, are usually lower compared to the assessment by an independent observer (affective fallacy bias) [20, 25]. However, to reduce confounding in the associations introduced by this bias, all results were adjusted for mental health status. Although some residual confounding may be expected as mental health status was quantified using one specific question, it is unlikely this analytical procedure explains the lack of association with MCS, as the VIF was low and excluding that variable from the analyses did not affect the statistical significance or direction of the associations (results not shown). Thirdly, 29% of the eligible participants refused to participate in the study. For ethical reasons, no any information was collected from these individuals, restraining us of assessing non-response bias. Nonetheless, the distribution of the evaluated sample according to sociodemographic variables is comparable to the available data for SA in the last Australian census [13]. Finally, causal inferences are not possible due to the cross-sectional design of this study.

## Conclusions

Chronic conditions are not only highly prevalent in the population, but they cluster in individuals. Furthermore, the relationship between multimorbidity and HRQoL is not only additive, as the coexistence of musculoskeletal conditions amplifies the association of other diseases with PCS. Our findings highlight the need for innovation in the management of patients with chronic health conditions, as active checking for comorbidities that cluster frequently may help identify individuals with reduced physical HRQoL. Therefore, it seems that a multidisciplinary approach might be more effective than focussing on single health conditions to improve HRQoL and promote healthy ageing. Further longitudinal studies will be necessary to elucidate the real impact of different clusters of chronic conditions on the different domains of HRQoL.

## Abbreviations

95%CI: Confidence intervals of 95%
HRQoL: Health-related quality of life
MCS: Mental component score
NCDS: Non-communicable chronic diseases
p25-p75: Interquartile range
PCS: Physical component score
R^2^: Determination coefficients
SA: South Australia
SA1: Statistical Areas Level 1
SEIFA-IRSAD: Australian Socio-Economic Indexes for Areas Index of Relative Socio-economic Advantage and Disadvantage
SF-12v1: Medical Outcomes Study Short Form 12
VIF: Variance inflation factor
β: Regression coefficients
